# Exploring Gender Dimensions of Treatment Programmes for Neglected Tropical Diseases in Uganda

**DOI:** 10.1371/journal.pntd.0002312

**Published:** 2013-07-11

**Authors:** Heather Rilkoff, Edridah Muheki Tukahebwa, Fiona M. Fleming, Jacqueline Leslie, Donald C. Cole

**Affiliations:** 1 Dalla Lana School of Public Health, University of Toronto, Toronto, Ontario, Canada; 2 Uganda Ministry of Health, Vector Control Division, Kampala, Uganda; 3 Schistosomiasis Control Initiative, Imperial College London, London, United Kingdom; Dodowa Health Research Centre, Ghana

## Abstract

**Background:**

Gender remains a recognized but relatively unexamined aspect of the potential challenges for treatment programmes for Neglected Tropical Diseases (NTDs). We sought to explore the role of gender in access to treatment in the Uganda National Neglected Tropical Disease Control Programme.

**Methodology/Principal Findings:**

Quantitative and qualitative data was collected in eight villages in Buyende and Kamuli districts, Eastern Uganda. Quantitative data on the number of persons treated by age and gender was identified from treatment registers in each village. Qualitative data was collected through semi-structured interviews with sub-county supervisors, participant observation and from focus group discussions with community leaders, community medicine distributors (CMDs), men, women who were pregnant or breastfeeding at the time of mass-treatment, and adolescent males and females. Findings include the following: (i) treatment registers are often incomplete making it difficult to obtain accurate estimates of the number of persons treated; (ii) males face more barriers to accessing treatment than women due to occupational roles which keep them away from households or villages for long periods, and males may be more distrustful of treatment; (iii) CMDs may be unaware of which medicines are safe for pregnant and breastfeeding women, resulting in women missing beneficial treatments.

**Conclusions/Significance:**

Findings highlight the need to improve community-level training in drug distribution which should include gender-specific issues and guidelines for treating pregnant and breastfeeding women. Accurate age and sex disaggregated measures of the number of community members who swallow the medicines are also needed to ensure proper monitoring and evaluation of treatment programmes.

## Author Summary

This study explored gender-related factors that may influence community member access and adherence to treatment programmes for NTDs in Uganda. A large number of previous studies have identified community-based mass-treatment programmes as an effective strategy to treat affected populations. However, limited evidence is available to discuss challenges to treatment access, adherence, delivery and monitoring at community level. Quantitative data from treatment registers suggested that men were less likely to access treatment than women in at least two villages. It also revealed difficulties in community-based monitoring of the programmes, creating challenges in ascertaining how many persons are able to access the programme. Qualitative data collected from district health workers, community leaders, community medicine distributors and community members suggested that socio-behavioural and structural barriers to treatment access may be present for both genders. Results of the study identify gender-based challenges to treatment access that should be considered in planning, implementing and evaluating national treatment programmes for NTDs.

## Introduction

Neglected Tropical Diseases (NTDs) are a group of parasitic, viral and bacterial diseases that affect at least a billion people worldwide [1], [2]. Predominantly seen in rural and underserved communities in Africa, the Middle East and Southeast Asia, NTDs can pose significant health implications for both male and female populations. While social and occupational roles may place men at an increased risk of acquiring certain NTDs, such as schistosomiasis [3], NTDs also have significant implications for female populations [4]. Schistosomiasis can cause pregnancy complications [4], [5], [6] and an increased risk of HIV transmission in women especially due to the presence of genital lesions [7], [8]. In addition, stigma from NTDs such as onchocerciasis can significantly affect female populations by causing disfigurement, which may in turn affect marriage prospects [9].

A recent increase in international advocacy and subsequent funding has led to the establishment of national programmes to treat NTDs in Africa, Asia and Latin America. The current strategies to treat NTDs through these national programmes have largely focused on mass drug administration (MDA), either through school-based treatment of children between the ages of 5–14 or through community-based treatment programmes [10], [11]. In the latter, community medicine distributors (CMDs) from Village Health Teams (VHTs) are selected by community members, who are then trained to distribute the drugs to the endemic community in conjunction with health education [10], [11]. Community treatment usually involves either a house-to-house distribution strategy, where CMDs visit each household in the community to distribute medicines, or centralized distribution, where community members gather in a central location and receive treatment from the CMD on specific treatment days [10].

Although these strategies are considered successful in treating affected populations, there is currently very little information on how gender may influence knowledge, perceptions and access to NTD treatment programs. Three studies [12], [13], [14] were identified which provided quantitative gender-stratified data on adherence to a community MDA programme. Clemmons *et al* [12] and Brieger *et al* [13] focussed on community directed treatment with ivermectin (CDTI) for onchocerciasis control, which follows a slightly different and more inclusive model of community participation than Uganda's main current MDA strategies of implementing either in April or October as directed by the Ministry of Health. In CDTI, community members select their own drug distributors and choose the time of year in which the distribution takes place. This requires coordination with the centralized distribution programme staff in order to ensure the availability of drug treatments at different times of the year based on community preference [15]. The integrated MDA programmes that have been implemented in Uganda and other countries may or may not necessarily involve community-selected drug distributors, and the time of distribution was set to coincide with other community health initiatives, such as Child Health Days, in order to take advantage of the existing immunisation and bednet distribution programmes [16]. Kamara *et al* [14]' s study of the Sierra Leone NTD programme found that the majority of districts did not have a gender imbalance in treatment coverage, but that males were significantly more likely to have received treatment in three out of the 13 surveyed districts. Additionally, although the World Health Organization (WHO) has recently created a database with information on MDA coverage rates by disease and country, the data sets are not disaggregated by gender, nor do they separately identify the adult and adolescent populations [17]. Coverage validation surveys of NTD programmes conducted by the US Centre for Disease Control (CDC) collect gender specific information on reported treatment uptake, but data presented in published reports does not show coverage rates by gender [18].

The majority of existing literature on gender and NTDs focuses on female populations. In a study of community perceptions of intestinal schistosomiasis, Anguzu *et al.* [19], note that the disadvantaged socio-economic status of women within rural communities in Uganda can prevent them from actively participating in health programmes, and from accessing information on control or preventative measures for the disease. However, Clemmons *et al* [12] note that although women are often less involved in the decision-making processes in community treatment programmes for onchocerciasis, they tend to identify themselves as more susceptible to the disease and express stronger feeling of the benefits of the programme, which may impact their long-term adherence with the programme.

Very little information exists on the participation of pregnant and breastfeeding women in treatment programmes. According to international guidelines established by WHO, praziquantel, used for treatment of schistosomiasis, is safe for pregnant and breastfeeding women; albendazole, used for treatment of soil transmitted helminths (STH) and, when combined with ivermectin, lymphatic filariasis, is safe for pregnant women after the first trimester and for breastfeeding women; ivermectin, which is used to treat the aforementioned lymphatic filariasis and onchocerciasis, should not be provided to pregnant women but is safe for breastfeeding women once the child is a month old; and Zithromax, used to treat trachoma, should not be provided to pregnant or breastfeeding women until the child is one year old [20]. Previous studies have noted that pregnancy may leave large numbers of women untreated if mass treatment takes place during the time of their pregnancy or lactation, and later treatment opportunities are unavailable or unknown [21]. However, no studies have been identified that discuss how integrated treatment programmes, which contain multiple drugs and more complex eligible and ineligible requirements, may further affect access and adherence to treatment among pregnant and breastfeeding women.

Very minimal published research is available to assess male participation in NTD programmes. Including male perspectives in gendered analyses of MDA programmes is particularly significant given that a growing body of literature suggests that men have lower adherence and higher default rates with other types of vertical treatment programmes such as for HIV [22] and tuberculosis [23]. Finally, information on adolescent access and adherence to treatment is scarce. One study examining CDTI for onchocerciasis in Cameroon, Nigeria and Tanzania, briefly mentions that young women were the least likely to be aware of how to prevent and treat onchocerciasis in comparison to older men and women [12].

The specific *objectives* of this study were (i) to explore gender-related factors which may influence participation in treatment programmes for NTDs in Uganda in adolescent and adult populations; (ii) to examine factors which may contribute to a gender bias in the treatment programme; and specifically, (iii) to identify whether pregnancy and breastfeeding presents a barrier to the participation of women in MDA.

## Methods

### Ethics Statement

Ethical clearance for the study was obtained from the Uganda National Council of Science and Technology and the University of Toronto Research Ethics Board. District health authorities, sub-county health officers, and community leaders were informed of the study and provided verbal consent prior to the study's commencement. Community leaders who participated in a focus group discussion (FGDs), as well as sub-county supervisors and CMDs, gave written consent. As literacy rates tended to be low within the districts, all other adult and adolescent focus group participants provided verbal consent prior to the commencement of the FGDs. The names of all participants were recorded in a register book prior to the commencement of each FGD, and verbal consent was recorded by the researcher beside the participant's name. Written or verbal consent was also obtained from the parents of adolescents under 18 years of age. Both the Uganda National Council of Science and Technology and the University of Toronto Research Ethics Board approved the use of verbal consent for community members due to potential literacy issues that might prevent written consent from being obtained.

### Methodological Approach

This study employed a mixed methods approach, using quantitative data from community registers on the number of persons treated, as well as qualitative data in the form of FGDs and key informant interviews. Initially, the study proposed to use both quantitative and qualitative data to triangulate findings. However, significant challenges were experienced in collecting quantitative data: in five of the eight villages sampled, community registers were incomplete, leaving only three villages with data that had been recorded in a useable way. As a result, data analysis was carried out using what Creswell [24] describes as a “Concurrent Embedded Strategy”. In this approach, analysis of the available quantitative data provided additional insights into the qualitative findings.

### Study Area

This study was carried out in Wankole and Kidera sub-counties of Kamuli and Buyende Districts respectively, in Eastern Uganda, which were both part of Kamuli district prior to the creation of Buyende as a separate district in 2010. The NTD control programme in both districts is implemented through the coordination of Kamuli District Vector Control Office. These sub-counties were purposively selected to capture a diversity of occupations, village sizes, and accessibility to town and city centres: Kidera, a remote area approximately 50 km north of Kamuli town centre which borders Lake Kyoga, is composed primarily of fishing villages, and Wankole, which lies approximately 35 km between Kamuli town and the city of Jinja, borders swampland and is primarily agro-based, with rice, maize, and sugarcane as the major crops. Two parishes were randomly selected from each sub-county, and from each parish, two villages were randomly selected, creating a total sample of eight villages.

### Data Collection Methods

Quantitative data were extracted in each village from the national NTD control programme registers of the number of persons treated in each household, their sex and age, and the number of tablets received. Additionally, population data from Village Household Register Books were gathered and matched to the NTD treatment registers by name, sex, and age to assess under or overestimation of those treated.

Qualitative methods included FGDs with adult men and women, adolescent males and females, community medicine distributors and community leaders in each village. Key informant interviews were also held with sub-country programme staff (Table 1). Focus group and interview guides were informed by Rathgeber and Vlassoff's [25] “Gender Framework for Tropical Diseases Research” and included questions on knowledge and beliefs about the diseases and the treatment programme, perceptions of risk, utilization of health services and the mass drug administration programme specifically, decision-making power and challenges faced in accessing the programme. The guides were developed in English and translated into Lusoga by a Ugandan research assistant, and subsequently back translated into English by a staff member at the Uganda Ministry of Health. The guides were then pretested in Jinja District, Uganda, and revised, according to pilot feedback, for use in the field.

**Table 1:** Total number of focus groups and study participants, Buyende and Kamuli districts, Uganda 2011.

Group	Number FGDs	Total Participants
Community Leaders^1^	8	30
Community Medicine Distributors	8	17
Adult Males	8	68
Adult Females *(Pregnant at time of Distribution)*	8	91
Adult Females *(Breastfeeding at time of Distribution)*	8	100
Adolescent Males	8	73
Adolescent Females	8	64

1 *Includes members of the Local Council 1 and Religious Leaders.*

FGDs were moderated in Lusoga by a Ugandan research assistant. Notes were taken of participants' verbal and non-verbal communication. Convenience sampling was used to select participants, with community leaders assisting in identifying 6–12 participants for each focus group (Table 1).

A total of two key informant interviews were conducted at sub-county level, one with each supervisor of the NTD programmes in Kidera and Wankole, respectively, to gain insights into the successes and challenge of the programme, any noted gender biases, and whether training in gender issues has been incorporated into the programme. Participant observation of the MDA was carried out in two communities in Wankole which were carrying out mass-treatment at the time of data collection. Field notes were recorded to document these visits. Data collection was carried out from June–August 2011, with approximately four weeks spent in each of the two districts.

### Data Analysis

Quantitative data were cleaned, but the planned analysis of gender and age relative to treatment status was restricted to three villages due to concerns with data validity. Using treatment registers kept by the CMDs in each community, the proportion of eligible males and females treated in each village was calculated. To validate the accuracy of the treatment registers, Village Household Register books, which provide a census of persons living in each village, were obtained from the Local Council Chairperson of each village. Proportions of persons treated were recalculated using the number of eligible males and females from the Village Household Register Books as the denominators.

FGD and interview data were recorded and transcribed into English. Qualitative data were analyzed with NVivo 8.0 software using thematic content analysis. Codes and sub-codes were developed through repeated scanning of the transcripts, which were then grouped into three thematic areas described in the Results below.

## Results

Each of the eight villages was actively administering MDA, however, most had not completed distribution of all the drugs for the treatment year. While most villages were expected to complete distribution in May 2011, by late June and early July only four villages had done so. Typically, a schedule of three courses of medication is followed, with separation of at least two weeks in between each medication to prevent interactions: first albendazole and ivermectin are distributed together, followed by praziquantel (if available), and finally Zithromax after several more weeks. In Wankole, the CMDs in each village reported distributing ivermectin, albendazole and Zithromax. However, the drugs that the CMDs reported distributing in Kidera varied: CMDs in one village reported distributing ivermectin, albendazole and Zithromax; CMDs in a second village reported distributing praziquantel, ivermectin, albendazole and Zithromax; in a third one, praziquantel, albendazole and Zithromax; and in a fourth village, only Zithromax. After conversations with the sub-county supervisor and CMDs in Kidera and an examination of the community treatment register books, it was unclear why certain communities may not have distributed ivermectin or albendazole. Annual shipments of praziquantel had been delayed in Kampala in 2011, and thus communities who distributed praziquantel were relying on remaining tablets from previous years MDA campaigns.

### Number of Persons Treated under the NTD Programme

Accessing valid quantitative data from each of the selected villages was challenging. Although all CMDs had a written record of who had received treatment in a treatment register, record keeping varied in quality. Of the four villages which had completed the annual mass treatment, the CMDs had not registered community members prior to mass treatment, and it was therefore difficult to ascertain if the entire community had been treated. In one of the four villages, very little information was recorded, and thus this village, as well as three other villages which had not completed distribution, were removed from the quantitative analysis. The treatment registers also did not contain important information such as the reasons for treatment refusal. The findings in Table 2 present results for the three villages where treatment had been completed, and thus where data on the numbers treated were also presumed to be complete. However significant discrepancies were noted when the total numbers eligible for treatment from the treatment registers were compared with the total numbers registered in the official Village Household Register Books (Table 2). Despite these challenges, two of the three villages consistently showed a higher proportion of females treated than males treated. Given that some females were also not eligible for treatment due to pregnancy, the gendered differences in eligible numbers treated were likely underestimated.

**Table 2:** Persons treated in the NTD programme^1^.

Village	Group (age range in years)	Total # of Eligible* Persons Treated, Treatment Register†	Total # of Eligible* Persons Listed Treatment Register†	Total # of Eligible* Persons Listed VHRB††	Proportion Treated with Treatment Register† Denominator	Proportion Treated with VHRB†† Denominator
Bugobi A	Women (18–49)	71	78	119	71/78 (91%)	71/119 (60%)
	Men (18–49)	45	58	202	45/58 (76%)	45/202 (22%)
Buyelo B	Women (18–49)	68	89	157	68/89 (76%)	68/157 (43%)
	Men (18–49)	64	88	114	64/88 (73%)	64/114 (56%)
Itamia Meeru	Women (18–49)	66	69	137	66/69 (96%)	66/137 (48%)
	Men (18–49)	46	52	124	46/52 (88%)	46/124 (37%)

1 Total persons treated according to NTD Control Programme treatment registers and Village Household Register Books (using available data sources). Of the eight villages in which the study was conducted, only the above three villages had a treatment register that appeared complete enough to use for analysis.

* Persons eligible for treatment include persons ages five and older who are not seriously ill (for praziquantel), persons ages five and older who are not seriously ill, pregnant or breastfeeding (for ivermectin and zithromax), or persons ages 1 and older who are not seriously ill, including pregnant women in their second or third trimester (for albendazole).

† Treatment registers are kept by the CMDs in each community and are used to register participants who are eligible to participate in the NTD programme.

†† VHRB is Village Household Register Book which is kept by the Chairman of the Local Council (the Village Level government structure) in each village, and registers the number of persons living in each village (including births, deaths, new migrants, etc.) including age and gender.

### Qualitative Findings

#### Problems with the distribution strategy

As indicated by the quantitative findings, challenges were noted with the community-based distribution strategy which seemed to inadvertently influence gendered differences in treatment access. In seven of the eight villages surveyed, a house-to-house distribution strategy was used whereby CMDs visited each household to distribute the medicines. Participant observation noted that the house-to-house distribution strategy was placing an undue burden on the CMDs as they must not only visit every household (which can be a challenge in and of itself, especially in remote communities where distances between households are far), but also revisit households in an attempt to find men and other household members who were not home during the first visit.

Data collected from FGDs and participant observation suggested that men may have a particularly difficult time accessing treatment from a house-to-house distribution strategy, as they may spend little or no time at home during day due to occupational roles such as farming, trading or truck driving which take place away from the household. Women, who typically spend at least part of their day at home in order to take care of domestic chores, appeared more likely to receive treatment, and, in turn, may have more knowledge of the programme due to their contact with the CMDs.

Another frequently mentioned challenge of the house-to-house distribution strategy was that community members were often unaware of when the CMDs will reach their houses. This created further problems, since those who are often away from home cannot plan to stay home to receive the treatment. During participant observation, it was also revealed that many women had not yet eaten before the treatment was brought, and were reluctant to take the treatment on an empty stomach for fear of side effects.

Finally, although guidelines from the Uganda Ministry of Health's NTD control programme state that health education on NTDs and the treatment programme should be provided to community members by the CMDs and their supervisors, very few participants stated that they had received any sort of health education. Very few CMDs mentioned that they had received training on causes and transmission of NTDs, and several CMDs reported that they had insufficient training to answer community members' questions about the diseases. Community leaders, CMDs, and community members frequently mentioned that the lack of health education was fuelling opposition to the programme from community members who did not know the intended purpose of the treatment programme:

*“Ignorance is bad because if you are ignorant you stay not informed at all. Whatever comes you just oppose; however, if you are informed, you cannot keep on opposing the programme.”*

*-Community leader, Kidera sub-county*

#### Attitudes towards the treatment programme

Findings from FGDs suggested gendered differences in attitudes towards the treatment programme. Most communities mentioned previously held rumours about harmful effects of the treatment which were particularly prevalent among males. However, only three communities stated that the rumours about the treatment were still pervasive among the majority of community members. In these communities, men and adolescent boys were described as more likely to be the purveyors of rumours about the treatment, as well as to “challenge” the CMDs, to ask difficult questions about why they should take the treatment, or to refuse treatment due to “not being sick”:

*“Those tablets were given out when I was away but the information I heard was that these tablets can harm the body in various ways.”*

*-Male, Wankole sub-county*

CMDs often expressed concerns and frustrations that they could not adequately address many of the difficult questions that the men posed, such as about side effects and long term consequences of taking the treatment. In several communities, community leaders and men also commented that they felt the CMDs were not informed enough about the medicines and the programme, and requested that “experts” be brought in to better inform them on the reasons for taking the drugs and provide detailed explanations of how the diseases were caused and how to prevent them.

In contrast, women were found to be more accepting of the programme and more willing to accept and adhere to the advice and information provided by the CMDs. Nevertheless, a notable divergence was found in one community in Kidera sub-county where men were deemed to be accepting of the programme, but women were described as fearful of receiving treatment. In this community, women's concerns were reported to be the result of fears of the VHT because the women claimed that children had died following immunizations that the health workers provided. However, in no other communities did women express concerns with the integrity of their community-level health workers; CMDs were generally perceived by both men and women as “good people”, who, for various reasons, were sometimes unable to carry out their duties fully.

Notable differences were also seen between adolescent boys and girls in terms of socio-behavioural influences on decisions to receive treatment. Conversations with community leaders and male and female adolescents suggested that adolescent girls were more likely to accept the treatment if parents felt positively about the programme. Conversely, adolescent boys may be more easily influenced by their peer groups, or by adult men in the community. Several adolescent boys stated that they would be more receptive to the treatment if other adolescents were trained to distribute drugs and answer their questions.

#### Barriers facing pregnant women

In seven of the eight communities surveyed, CMDs expressed confusion or presented information that was contrary to that provided in the Uganda NTD Training of the Trainer's (ToT) Manual about which drugs should be provided to pregnant and breastfeeding women. The ToT manual states that pregnant women who are passed their first trimester should receive albendazole, and all women should receive praziquantel. Zithromax and ivermectin should not be provided to pregnant women. In five villages, CMDs stated that pregnant women should not receive any drugs at all. This suggests that many women are missing beneficial treatments for schistosomiasis and STH. In two villages, CMDs stated that women should receive Zithromax, a drug currently not recommended for pregnant or breastfeeding women until the child reaches one year of age.

Given the unreliable information provided by the CMDs, it is unsurprising that pregnant and breastfeeding women expressed confusion as to which drugs they were supposed to take. At least half of the participants in each focus group were unaware of when they were eligible to receive treatment after they had delivered their child. Sub-county supervisors stated that women did not come to the health centre to access treatment after giving birth. Participants were often unaware that treatment was available at health centres and could be accessed once they were again eligible to receive treatment, and had instead waited for the treatment to be delivered during the next treatment round. Many women reported missing mass treatment at least twice, and several had missed three or four times due to being pregnant or breastfeeding at the time of distribution. Some women expressed fears at the number of times they had missed treatment:

*“This medicine I do not know whether I will get chance to get it because I have missed three times, the first time I was pregnant, the second time I was breast feeding, this year I am pregnant now, so I don't really know. These diseases are bound to attack me, I am sure.”*

*-Pregnant Woman, Wankole sub-county*

## Discussion

Our findings reflect some of the challenges for MDA programmes in treating NTDs with regards to monitoring drug coverage and ensuring that all eligible persons are able and willing to access treatment. Our quantitative findings provide some suggestion that men are less likely to receive treatment than eligible women. Because the quantitative denominator data was taken from both NTD programme treatment registers and household register books from the villages, it is most likely that this reflects the number of men who are actual residents of the village, rather than temporary migrants, as has been found in other studies [26]. Findings also indicate programmatic challenges and illustrate the difficulties in assessing treatment coverage rates at a community level. Similar findings were identified by Parker and Allen [26] who observed significant problems with the community NTD control programme registers in some areas, which were often missing or incomplete, creating difficulties in estimating the number of persons treated each year. This is in contrast, however, to a larger coverage study by Kabatereine *et al.* [27] who found that community medicine distributors were at least moderately accurate with record keeping, with approximately 61% of communities having entered data correctly in their registers. Although the study by Kabatereine and colleagues [27] focused on mass treatment for schistosomiasis and STH only, it nonetheless highlights several areas of concern regarding the community-based strategy for drug distribution, including inadequate registration of community members in certain areas, which may result in shortages of medicines and low treatment coverage.

Our qualitative findings were able to shed light on specific social, behavioural and programmatic issues which are creating and reinforcing gender differences in access to treatment. Unlike Anguzu *et al.* [19] and Clemmons *et al.* [12], our findings do not suggest a bias towards males in terms of access to information or decision-making power regarding treatment access. This may be due to social and cultural differences between Anguzu *et al.* (carried out in Busia District, East Uganda) and Clemmons *et al.* (carried out in Cameroon, Tanzania and Nigeria) study populations and the present study setting, or to differences in family structure and hierarchy. Alternately, this may simply be a consequence of male absence when the CMDs arrive to distribute treatment, which leaves females with more decision-making power and provides them with an opportunity to ask CMDs questions about the treatment. However, our findings suggest socio-behavioural and structural barriers are present for both men and women, but tend to differ between the genders. The majority of villages in this study relied almost exclusively on a house-to-house distribution strategy which appeared to be particularly unsuitable for accessing males. Whether such gender-specific barriers are also present in a centralized distribution strategy, where community members gather in a central place on specified days to receive treatment, should be explored in future research.

Findings of this study also support previously published work suggesting that access to treatment is particularly difficult for pregnant and breastfeeding women [21]. This is significant given the potential for pregnant women to suffer negative consequences of NTDs during pregnancy. Several studies have identified anthelminthic treatments during pregnancy as having positive outcomes on reducing maternal anemia [28], [29], [30], although this benefit may be realised only in women with moderate to high intensity helminth infections [31], [32]. The potential for additional health gains by reducing low birth weights and infant mortality through anthelminthic treatments have also been noted in observational studies [30]. In addition, treating pregnant women who are co-infected with malaria and HIV for the STH can reduce the intensity of malarial infection [33], and may benefit those with a higher viral load [34]. Finally, it is estimated that women living in schistosomiasis endemic areas may spend up to 25% of their reproductive years pregnant and another 60% of this time lactating [35]. Thus, as indicated by our results, women who miss treatment due to pregnancy and breastfeeding are likely to repeatedly miss treatment, and may be more susceptible to organ damage and cancer due to chronic schistosomiasis infection [35].

The lack of access to treatment by pregnant and breastfeeding women and the health risks resulting highlight the need to improve community knowledge that medicines are available at health centres following the community-based treatment period, a point that was previously raised in a process evaluation carried out in Uganda in 2006 [16]. Greater training for CMDs on guidelines for treating pregnant and breastfeeding women, and encouragement of health workers to provide medicines for women attending postnatal clinics may also help improve access for this population. However, further research and monitoring of adverse outcomes is also necessary as recent studies have suggested that albendazole and praziquantel use during pregnancy may increase risk of infantile eczema [31] and may also have positive or negative effects on the development of the fetal immune system and disease susceptibility later in life [32].

**Figure 1:**
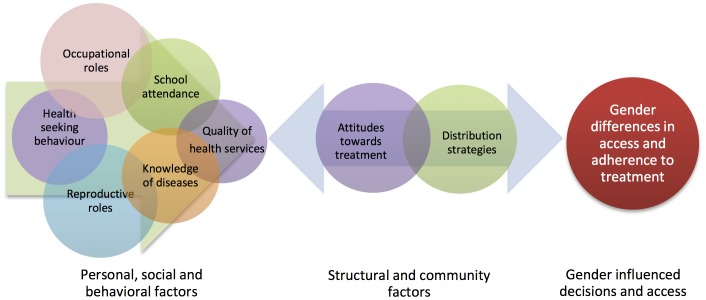
Framework for gendered differences in access and adherence to NTD treatment programmes.

The use of convenience sampling and the limited number of villages included in the sampling frame, while appropriate for the qualitative methods used, does not allow for generalizability to other areas of Uganda. Thus, the extent and type of gender bias present in the sampled villages cannot be compared to other villages or regions in Uganda. Instead, the combined results of the quantitative and qualitative findings provide insights into some of the broad issues that may influence gendered difference in access and adherence to treatment. Figure 1 provides a framework which synthesizes the information from qualitative and quantitative findings as follows: personal circumstances and social and behavioural aspects, which are often gender specific, create and reinforce personal and community attitudes towards the treatment programme. Attitudes towards the treatment programme may create problems with the distribution strategy if community members are unable or unwilling to benefit from the programme. Additionally, problems with the distribution strategy, such as insufficiently-trained CMDs and lack of health education, reinforce negative attitudes towards the programme, as CMDs are unable to refute rumours about the treatment or convince community members of the importance of the programme. Moreover, specific programmatic decisions, such as the annual house-to-house distribution strategy which tends to miss adult males and pregnant women, also contribute to the large number of persons who are apparently missed during mass-treatment.

Rathgeber and Vlassoff's [25] “Gender Framework for Tropical Diseases Research”, which was used to inform data collection tools, provides a comprehensive and integrated synthesis of social, economic and personal factors which are contributing to gendered differences in tropical diseases. This includes social and behavioural factors at the personal level and the community level as well as at the policy level that affect access and use of health services. However, since this framework was developed prior to the initiation of national NTD programmes, its authors did not consider critical programmatic factors such as challenges with the distribution strategy in accessing men and pregnant women. This framework is also limited by its inability to elucidate the direction of causality of these social, economic and personal factors, which would be helpful for refining future NTD programming.

Figure 1 attempts to build on Rathgeber and Vlasoff's framework by placing community attitudes and problems with the distribution strategy at the centre of the issue, arguing that a more comprehensive understanding of the nuances and challenges of community-based treatment programmes is desperately needed to address gender-related challenges and ensure future success of the programmes.

Our results have several implications for community-level programming. In communities where coverage rates are low, there is likely a need to augment the training of community personnel. Rumours about the treatment, the inability of CMDs to answer community member's questions, as well as negative experiences with a previous community immunization programme, can all affect attitudes towards the treatment, and can have important consequences for adherence to the NTD programme. Thus, the capacity of CMDs to facilitate health education, answer community members' questions about treatment, understand the causes of diseases, and manage side-effects caused by the medicines must be strengthened. Brieger *et al.* [13] have suggested holding ongoing refresher workshops for the CMDs to support them in addressing problems with adherence within their communities. Such workshops could emphasize the importance of the CMDs as motivators of others to take the treatment, and provide in-depth information on side effects of medications and the causes of the diseases. To address problems with the distribution strategy, CMDs should also be encouraged to consult with groups who spend long periods outside of the village and determine an optimal time and strategy for reaching these groups when they are home. They should also be encouraged to discuss these issues with sub-county program staff so strategies for reaching groups with low adherence rates can be developed, and programmes can be modified accordingly.

Finally, there is a need to work with communities to improve self-monitoring and evaluation strategies. However, it should be recognized that it is time consuming for CMDs to travel to each individual household prior to mass-distribution to register village members, and that record-keeping also adds to the workload. In communities where household registration is not completed, or is completed inconsistently, it may make the most sense to encourage CMDs to ensure, at minimum, that treatment numbers are correctly recorded. Concurrently, district, national or international stakeholders should invest increased effort into coverage surveys and qualitative follow-up activities to provide more general validation and insight. Moreover, given the demands of distributing and monitoring drug coverage, the challenges with low coverage rates in some communities, the training and skills required of the CMDs on knowledge of diseases and treatments, and the considerable time that must be spent to troubleshoot methods of improving adherence, there may be a need to reconsider the voluntary status of the CMDs. We recognize that this is a controversial point, given that other work has noted that compensation does not necessarily contribute to higher coverage rates or lower attrition among CMDs [36]. While the results of our study are context-sensitive and cannot be generalized for the rest of Uganda, other research has also raised the point that the growing number of community-based programmes reliant on CMDs may take them away from their own income-generating activities, and thus create a need for monetary compensation [37]. It is therefore suggested that national programme evaluations examine CMD workload and attrition as a potential factor in communities with low coverage rates.

Our results also highlight the critical importance of CMDs in influencing community member adherence to the programme. Many of the challenges described are unlikely to be addressed unless NTD programme management are able to commit greater resources to mitigating community-level challenges. Resources required are those for ensuring quality training of CMDs, NTD-related health education and mobilisation for community members, and motivation for CMDs to carry out their duties efficiently and effectively.

Greater attention to community-level challenges is particularly important given the limitations in monitoring and evaluating coverage of the programme and its operations. According to a recent article by Research Triangle Institute (RTI) International, the main international agency overseeing the current integrated NTD programme, at least 98 million persons have been treated under the programme in the past three years [38]. Evidence of these findings is reported through self-report from drug distributors and their supervisors, which was then validated with subsequent coverage studies [38]. Our findings illustrate the important gender-related social, behavioural and personal factors that may affect community members' ability to access treatment. In addition to quantifying the number of persons by gender and age group who are taking the treatment, there is also a need for a greater understanding of why persons are choosing or not choosing to participate in mass treatment, and the context in which challenges with distribution or adherence are occurring. As Kabatereine *et al.* [39] note, funding for in-depth monitoring and evaluation is limited given that the majority of resources are currently directed towards intervention delivery which precludes the acquisition of the data needed to determine whether modifications to programmatic activities are needed to increase their effectiveness. Evaluations conducted at the national or international level could compliment community-level data collection, and should include gender and age disaggregated data, as well as additional qualitative or other research methods to understand in a more nuanced way the complex social, behavioural and structural issues that can affect coverage rates.

### Conclusion

Over the past decade, significant progress has been made in controlling or eliminating NTDs with billions of dollars devoted to treating the diseases through national programmes in sub-Saharan Africa and elsewhere. However, more resources must be devoted to understanding implementation at the community level to address the nuances and challenges of drug distribution within communities. Our study focused on gender issues and identified several key areas where modifications and increased monitoring at the community level may significantly improve access to treatment for both males and, especially pregnant females. Monitoring and evaluation strategies should include social and behavioural data in addition to accurate sex- and age-disaggregated data on the number of persons who are adhering and not adhering to the NTD programme. These challenges must be addressed if long-term goals of controlling NTDs are to be achieved.
